# Quantifying the mechanisms of domain gain in animal proteins

**DOI:** 10.1186/gb-2010-11-7-r74

**Published:** 2010-07-15

**Authors:** Marija Buljan, Adam Frankish, Alex Bateman

**Affiliations:** 1 Wellcome Trust Sanger Institute, Wellcome Trust Genome Campus, Hinxton, Cambridge, CB10 1SA, UK

## Abstract

**Background:**

Protein domains are protein regions that are shared among different proteins and are frequently functionally and structurally independent from the rest of the protein. Novel domain combinations have a major role in evolutionary innovation. However, the relative contributions of the different molecular mechanisms that underlie domain gains in animals are still unknown. By using animal gene phylogenies we were able to identify a set of high confidence domain gain events and by looking at their coding DNA investigate the causative mechanisms.

**Results:**

Here we show that the major mechanism for gains of new domains in metazoan proteins is likely to be gene fusion through joining of exons from adjacent genes, possibly mediated by non-allelic homologous recombination. Retroposition and insertion of exons into ancestral introns through intronic recombination are, in contrast to previous expectations, only minor contributors to domain gains and have accounted for less than 1% and 10% of high confidence domain gain events, respectively. Additionally, exonization of previously non-coding regions appears to be an important mechanism for addition of disordered segments to proteins. We observe that gene duplication has preceded domain gain in at least 80% of the gain events.

**Conclusions:**

The interplay of gene duplication and domain gain demonstrates an important mechanism for fast neofunctionalization of genes.

## Background

Protein domains are fundamental and largely independent units of protein structure and function that occur in a number of different combinations or domain architectures [1]. Most proteins have two or more domains [2] and, interestingly, more complex organisms have more complex domain architectures, as well as a greater variety of domain combinations [2-4]. A possible implication of this phenomenon is that new domain architectures have acted as drivers of the evolution of organismal complexity [3]. This is supported by a recent study that experimentally showed that recombination of domains encoded by genes that belong to the yeast mating pathway had a major influence on phenotype [5]. While there is evidence that in prokaryotes new domains are predominantly acquired through fusions of adjacent genes [6,7], determining the predominant molecular mechanisms that underlie gains of new domains in animals has been more challenging [3].

**Figure 1:**
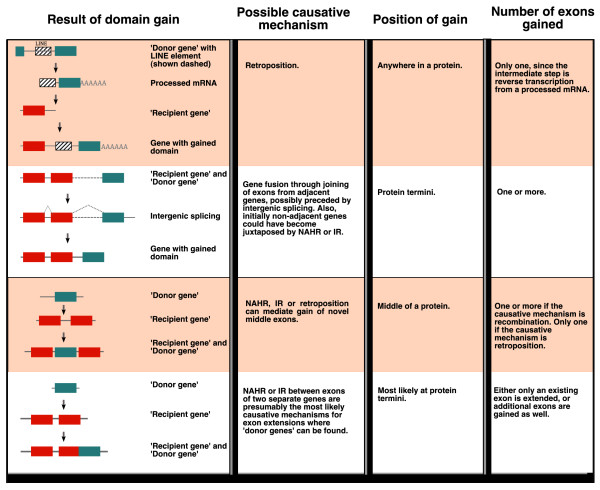
**Summary of mechanisms for domain gains**. This figure shows potential mechanisms leading to domain gains and the signals that can be used to detect the causative mechanism. Domain gain by retroposition is illustrated as an example where the domain is transcribed together with the upstream long interspersed nuclear element (LINE), but other means of retroposition are also possible [3]. The list of possible mechanisms is not exhaustive and other scenarios can occur, such as, for example, exonization of previously non-coding sequence or gain of a viral or transposon domain during retroelement replication. IR, illegitimate recombination; NAHR, non-allelic homologous recombination.

The question of what mechanisms underlie domain gains is related to the question of what mechanisms underlie novel gene creation [3,8,9]. The recent increased availability of animal genome and transcriptome sequences offers a valuable resource for addressing these questions. The main proposed genetic mechanisms that are capable of creating novel genes and also causing domain gain in animals are retroposition, gene fusion through joining of exons from adjacent genes, and DNA recombination [3,8,9] (Figure 1). Since these mechanisms can leave specific traces in the genome, it may be possible to infer the causative mechanism by inspecting the DNA sequence that encodes the gained domain. By using retrotransposon machinery, in a process termed retroposition, a native coding sequence can be copied and inserted somewhere else in the genome. The copy is made from a processed mRNA, so sequences gained by this mechanism are usually intronless and have an origin in the same genome. This was proposed as a powerful means for domain shuffling, but the evidence for its action is still limited [10,11]. Recent studies observed a phenomenon where adjacent genes, or nearby genes on the same strand, undergo intergenic splicing and create chimerical transcripts [12-14]. This suggested that if regulatory sequences between the two genes were degraded during evolution, then exons of the genes could be joined into a novel chimeric gene. As a consequence of this, one would observe a gain of novel exon(s) at protein termini. One example for this mechanism is the creation of the human gene *Kua-UEV *[15]. Recombination can aid novel gene creation by juxtaposing new gene combinations, thereby assisting exons from adjacent genes to combine. Alternatively, recombination could also occur between exonic sequences of two different genes [16]. The two main types of recombination are non-allelic homologous recombination (NAHR) [8,17], which relies on short regions of homology, and illegitimate recombination (IR) [8,9,18], also known as non-homologous end joining, which does not require such homologous regions. In addition to these mechanisms, new protein coding sequence can be gained through: 1, deletion of the intervening sequence between two adjacent genes and subsequent exon fusion [19]; 2, exonization of previously non-coding sequence [20]; and 3, insertion of viral or transposon sequences into a gene [21]. Interestingly, direct examples for any of these mechanisms are still rare.

Protein evolution has frequently been addressed by studying the evolution of domain architectures [22,23]. Specific examples in animals have been reported for domain gains through exon insertions into introns [24]. The extracellular function of these inserted domains indicates the importance of this mechanism for the evolution of multicellular organisms. Additionally, more recent whole-genome studies of domain shuffling have also focused on domains that are candidates for exon insertions into introns - for example, domains that are surrounded by introns of symmetrical phases [25-27]. These studies have suggested that domain insertions into introns - that is, gain of novel middle exons - have had an important role in the evolution of eukaryotic proteomes. The initial studies attributed intronic insertions to intronic recombination, and the more recent studies have also acknowledged the potential role of retroposition in this process.

In this work, we use the phylogenetic relationships between genes from completely sequenced metazoan genomes in order to address the question of what mechanisms underlie the gains of novel domains. To do this, we first identify a set of high-confidence domain gain events and then look at the characteristics of the sequences that encode these domains. Our results show that gene fusion through joining of exons from adjacent genes has been a dominant process leading to gains of new domains. Two other mechanisms that have been proposed as important contributors to gains of new domains in animals, retroposition and insertion of exons into ancestral introns through intronic recombination, appear to be minor contributors. Furthermore, we observe that most domain gain events have involved gene duplication and that domain gains often relied on DNA recombination. Based on the results presented here, we propose that these gain events were frequently assisted by NAHR, which played a role in creating gene duplicates and in the juxtaposition of the ancestral genes concerned.

## Results

### Set of high-confidence domain gain events

To find a set of high-confidence domain gain events, we used gene phylogenies of completely sequenced animal genomes from the TreeFam database [28]. TreeFam contains phylogenetic trees of animal gene families and is able to assign ortholog and paralog relationships because it records the positions of speciation and duplication events in the phylogenies. We assigned domains to the protein sequences in these families according to Pfam annotation [29]. The Pfam database provides the currently most comprehensive collection of manually curated protein domain signatures. Its family assignments are based on evolutionarily conserved motifs in the protein sequences.

It is important to distinguish real domain gain events from domain gain calls caused by errors in gene and domain annotations. To obtain a set of high-confidence domain gains, we implemented an algorithm that ensured that a gain is not falsely called when other genes in that family had actually experienced multiple losses of the domain in question. We also took into account only those gains that had at least one representative sequence in a genome of better quality and we discarded gains where there was only one sequence with the gained domain, that is, gain was on the leaf of the phylogenetic tree. We did this to overcome the issue of erroneous gene annotations. We then refined the initial domain assignments to find domains that were missed in the initial Pfam-based annotation and then discarded all dubious domain gain cases where there was evidence that a domain gain was called due to incorrectly missing Pfam annotations. After filtering for confounding factors that could cause false domain gain calls and taking into account only examples where the same transcript contains both the ancestral portion of the gene and a sequence coding for a new domain, we were left with 330 events where we could be confident that one or more domains had been gained by an ancestral protein during animal evolution - we took into account only gains of new domains, and not duplications of existing domains. The final set will not be comprehensive, but these filtering steps were necessary to ensure that we have a set of high-confidence domain gain events. Moreover, none of these steps introduces a bias towards any one mechanism over another. The only mechanism of domain gain that we cannot detect after this filtering is the case where amino acid mutations in the sequence created signatures of a novel domain that was not previously present in any protein; for example, when point mutations in the mammalian lineage created signatures of a mammalian-specific domain.

### Characteristics of the gained domains

To investigate which molecular mechanisms have caused domain gains in our set of high-confidence domain gain events, we examined the characteristics of the sequences that code for the gained domains. As a requirement, each gain event in our set has as descendants two or more genes with the gained domain. To simplify the investigation, we only considered one representative protein for each gain event, and most (232 or 70%) of these were drawn from the human genome as its gene annotation is of the highest quality. Sometimes the same protein was an example for more than one domain gain that occurred during evolution. We projected intron-exon boundaries and intron phases onto the representative protein sequences to help identify the possible causative mechanism. We also compared each representative protein sequence with the orthologs and paralogs in the same TreeFam family that lacked the gained domain. This helped us to assign the characteristics of the gained domains.

**Figure 2:**
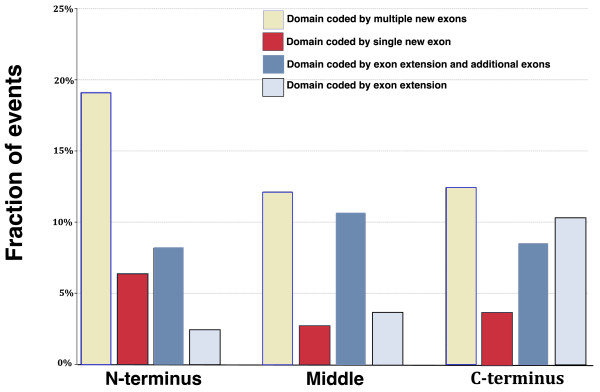
**Distribution of domain gain events according to the position of domain insertion and number of exons gained**. Gains at amino and carboxyl termini and in the middle of proteins are shown separately. The first column in each group shows the fraction of gains where the gained domain is coded by multiple new exons and the second where it is coded by a single new exon. The third column shows the fraction of gains where the ancestral exon has been extended and the gained domain is coded by the extended exon as well as by additional exons. Finally, the fourth column in each group shows cases where only the ancestral exon has been extended with the sequence of a new domain.

We recorded domain gain position (amino-terminal, carboxy-terminal or middle) as well as the number of gained exons and whether the domain was an extension of an existing exon (Figure 2). We observed two pronounced trends: first, most of the domain gains (234 or 71% of the events) occurred at protein termini. This was in agreement with previous studies [30,31], and terminal domains were significantly overrepresented among the gained domains (P-value < 7.7 × 10^-13^, Chi-square test; Additional file 1). Second, most of the gained domains (again 234 or 71%) are coded for by more than one exon and therefore retroposition is excluded as a likely causative mechanism for them. Figure 2 and evidence for other mechanisms of domain gain, including analysis of gain events that have possibly occurred through exonisation of non-coding sequences [21] and through inclusion of mobile genetic elements [32], is further discussed in Additional file 1.

Even though we do not expect that the final set of high-confidence domain gains is biased towards any of the mechanisms, the total number of gain events in the set is relatively small and this could introduce apparent dominance of one mechanism over another. Hence, we wanted to test whether a larger set of domain gains would support the observed distribution of characteristics of gained domains. We composed the larger (medium confidence) set by excluding two out of the three filtering criteria (Additional file 2a). We left only the criteria for domain gains to be supported by a gain in an organism with a better quality genome, because the distribution of domain gains that are reported only in one protein showed a bias towards the genomes of lower quality (the most gains were reported in *Schistosoma mansoni *and *Tetraodon nigroviridis *(320 and 303 gains, respectively), and among the organisms with least reported gains were human and mouse (25 and 19 gains, respectively)). We compared the high and medium confidence sets of gain events (Additional file 3). The distribution of domain gains in the medium-confidence set is overall similar to the one in the set of high-confidence domain gains, thus supporting the major conclusions we draw here. The major difference between the two sets was in the number of middle domains coded by one exon: there were 1.8 times more gains of a domain coded by a single novel middle exon, and 1.6 times more gains of a domain coded by an extension of a middle exon in the medium-confidence set. The set of medium-confidence domain gains is enriched with false domain gain calls caused by discrepancies in the domain annotation of proteins from the same TreeFam families. However, we cannot rule out that some of these gains are real; hence, more supporting cases for the mechanisms that can add domains to the middle of proteins could be found in the larger set. Mechanisms that could be at play here are retroposition and exonization of previously non-coding sequence, but also recombination inside the gene sequence.

We chose a single representative transcript for each gain event, but as a control we compared the characteristics of the gained domain in all descendant TreeFam transcripts with the gained domain. In most cases we found that other descendants of the gain event had the same characteristics of domain gain as the representative protein (in 76% of descendants of a gain event, on average). This suggests that the causative mechanism can be investigated by looking at the characteristics of the domain in one representative protein for each gain. Additionally, we tested whether deficiencies in the current transcript assignments introduce false domain gain calls and found that not more than 4% of domain gain calls could be due to discrepancies in gene annotations (Additional file 4) [33]. Hence, we expect that these domains will not influence the overall distribution of domain characteristics.

**Figure 3:**
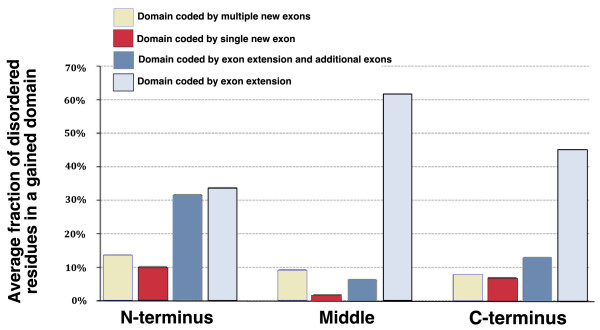
**Distribution of disordered residues in the gained domains according to the position of domain insertion and number of exons gained**. This graph shows the percentage of disordered residues in each category of domain gains. The fraction of events in each category can be seen in Figure 2.

We were intrigued by the many gains coded by exon extension. These domain gains are more likely to be enriched in domains gained through exonisation of non-coding sequences compared to other categories of domain gains. We would expect that when a new Pfam family is formed from previously non-coding sequence that it is more likely that this will be an intrinsically unstructured region. Intrinsically unstructured or disordered regions lack stable secondary and/or tertiary structure, but are associated with important functions, such as regulation and signaling [34-36]. We predicted disordered regions in all proteins from the study with the IUPred software [37] and looked at the average percentage of disordered residues in each gained domain in our set and in all other domains present in these proteins (Figure 3). We observed two prominent trends: first, gained domains in general have a greater percentage of disordered residues (on average, only 5% of residues of all other domains in proteins are predicted to be disordered compared to an average of 21% of residues in the gained domains); and second, domains with the greatest percentage of disordered residues are those that have been gained by extension of existing exons. These results suggest a link between the evolution of new unstructured domains and exonization of non-coding sequence.

### Donor genes of the gained domains

We investigated whether duplication of the sequence of the 'donor genes' preceded gains of these domains. We selected the 232 gain events with human representative proteins; the selected domain gain events cover those events where at least one of the descendants is a human protein. Hence, the time scale for these events ranges from the divergence of all animals (around 700 million years ago) to the divergence of primates (around 25 million years ago). We grouped descendants of each gain event into the evolutionary group (primates, mammals, vertebrates, bilaterates and animals) they span. Additional file 5 lists all gain events together with information about the evolutionary group of the descendants with the gained domain. For each domain, we checked whether any other human protein contains sequence stretch similar to the gained domain. When there is a sequence significantly similar to the gained domain somewhere else in the genome, it is possible that the original sequence was duplicated and that one copy was the source of the gained domain. For this we used Wu-blastp [38] and found a potential origin for 129 (56%) of the gained domains. For the remaining domains it is possible that either the mechanism for domain gain did not involve duplication of an existing 'donor' domain, or that the two sequences have diverged beyond recognition. Hence, the set of domains without the potential 'donor' is enriched in events where the domain has been gained through exonization of previously non-coding sequence, or, for example, through gene fusion without previous gene duplication.

### Evidence for the molecular mechanisms that caused domain gains

**Figure 4:**
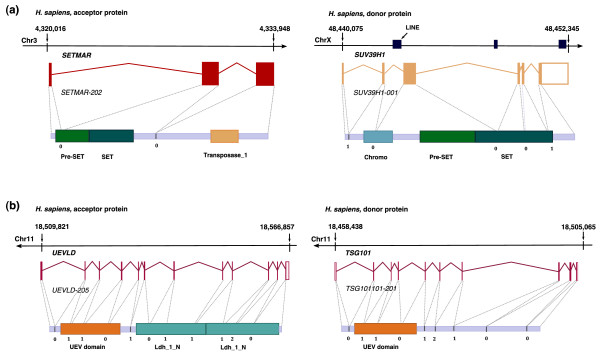
**Examples of evidence for mechanisms that have caused domain gains**. (**a**) An example of a domain gain mediated by retroposition. TreeFam family TF352220 contains genes with a transposase domain (PF01359). The primate transcripts in this family have been extended at their amino terminus with the pre-SET and SET domains. The representative transcript for this gain event is *SETMAR-201 *(*ENST00000307483*; left-hand side). Both gained domains have a significant hit in the gene *SUV39H1 *(*ENSG00000101945*; right-hand side) - the Set domains of the donor and recipient proteins share 41% identity. Previously, it has been reported that the chimeric gene originated in primates by insertion of the transposase domain (PF01359, with mutated active site and no transposase activity) in the gene that contained the pre-SET and SET domains [21]. Here we propose that the evolution of this gene involved two crucial steps: retroposition of the sequence coding for the pre-SET and SET domains and the already described insertion of the MAR transposase region [21]. The SET domain has lost the introns present in the original sequence and the pre-SET domain has an intron containing repeat elements in a position not present in the original domain, suggesting it was inserted later on. The likely evolutionary scenario here includes duplication of pre-SET and SET domains through retroposition, insertion of the transposase domain and subsequent joining of these domains. The *SETMAR *gene is in the intron of another gene (*SUMF1*), which is on the opposite strand, so it might be that *SETMAR *is using the other gene's regulatory regions for its transcription. The top of the figure shows the genomic positions of depicted genes. Arrowheads on the lines that represent chromosomal sequences indicate whether the transcripts are coded by the forward or reverse strand. Transcripts are always shown in the 5' to 3' orientation and proteins in the amino- to carboxy-terminal orientation. Exon projections and intron phases are also shown on the protein level. Pfam domains are illustrated as colored boxes. Figure 4b and Additional file 8 use the same conventions. (**b**) An example of a domain gain by gene duplication followed by exon joining. TreeFam family TF314963 contains genes with a lactate/malate dehydrogenase domain where one branch with vertebrate genes has gained the additional UEV domain. Homologues, both orthologues and paralogues, without the gained domains are present in a number of animal genomes. A representative transcript with the gained domain is *UEVLD-205 *(*ENST00000396197*; left-hand side). The UEV domain in that transcript is 56% identical to the UEV domain in the transcript *TSG101-201 *(*ENST00000251968*), which belongs to the neighboring gene *TSG101*, and the two transcripts also have introns with identical phases in the same positions. The likely scenario is that after the gene coding for the *TSG101-201 *transcript was duplicated, its exons were joined with those of the *UEVLD-205 *ancestor and the two genes have been fused.

Domains in the human lineage for which we can identify a potential donor protein and that are gained within a single exon are possible candidates for retroposition (26 cases). We checked these cases manually and found that only one of them was plausibly mediated by this mechanism (Figure 4a); the pre-SET and SET domains in the *SETMAR *gene were most likely gained by retroposition and have an origin in the gene *SUV39H1*. Interestingly, this gene lies within the intron of another gene on the opposite strand, which implies a possible means for overriding the need for the evolution of novel regulatory signals. A similar observation has been reported for the examples of evolution of novel human genes [39]. The other 25 cases lacked supporting evidence for this mechanism (Additional file 6) [40-42]. The lack of evidence is not a definite proof that retroposition was not the active mechanism. However, over 70% of the gained domains in the whole set are coded for by more than one exon, and even though some of the retroposed sequences can acquire introns later on, intron presence in the majority (234) of the gained domains rules out retroposition as a likely widespread mechanism of domain gain. Moreover, a number of possible candidates for a gain by retroposition in the human lineage are better explained by joining of exons from adjacent genes. With regard to other lineages, only the gains in insects, with representative proteins from *Drosophila melanogaster*, have numerous examples (22 cases) of a gain of domain coded by one exon, leaving open the possibility that retroposition might be a more important mechanism for domain gain in insects than it is in other lineages. However, overall this seems to be a rare mechanism for domain gain in animals and there are also indications of the importance of adjacent gene joining [11] and NAHR [43] in the formation of chimeric genes in the *Drosophila *lineage.

Terminal gains of domains coded by multiple novel exons are particularly interesting here because for these events there is only one plausible causative mechanism: joining of exons from adjacent genes (Figure 1). Even though, because of the criteria we used, the number of new exons gained at termini is a lower estimate, this is still the most abundant type of event; 104 (32%) of all events are amino-terminal (63 events) or carboxy-terminal (41 events) gains of domains encoded by multiple new exons (Figure 2). We can discard retroposition and recombination assisted insertions into introns as likely mechanisms for these gains. However, it is possible that recombination preceded domain gains, and even that recombination did not juxtapose fully functional genes but only, for example, certain exons of one or both of the genes. Indeed, we have not found that these genes exist as adjacent separate genes in the modern genomes (Additional file 7) [44] and it is likely that these gains were preceded by DNA recombination.

The search for the 'donor gene' of the gained domains identified the possible origin of the domain for 60% of domains encoded by new terminal exons. This implies that duplication of a donor domain has frequently provided the material for subsequent exon joining and new exon combinations. An illustration of this mechanism is the gain of the UEV domain in the *UEVLD *gene (Figure 4b). The gain has most likely occurred after the neighboring gene *TSG101 *has been duplicated and exons of one copy joined with exons of the *UEVLD *ancestor. Two similar examples are illustrated in Additional file 8a,b.

Because of the special attention that has been given to domain insertions into introns in discussions on domain shuffling during protein evolution [26,40], we have studied the middle gains of novel exons in more detail (see also Additional files 6 and 9). Out of 49 domains encoded by novel exons and gained in the middle of proteins, 28 are surrounded by introns of symmetrical phases, and hence give further support to the assumption that the causative mechanism for them indeed included insertions into ancestral introns. However, these likely examples for domain insertions into introns cover less than 10% of all gain events, which does not support the expectation that this was the major mechanism for domain gains in the evolution of metazoa [25,26]. This is even more pronounced if we take into account the fact that when ancestral proteins are encoded by more than two exons, the possible number of insertions into the middle is higher than the possible number of insertions at the end of the protein [31]. It is also worth noting that most (82% or 40 of 49 intronic gains) domains inserted into ancestral introns were coded by multiple exons, which implies that intronic recombination, rather than retroposition, would be the more likely causative mechanism for the majority of intronic gains.

Gains in the representative human proteins illustrate the characteristics of domains that were gained during evolution of the human lineage. However, it is important to note that at different stages of evolution, different mechanisms could have predominated. The same is true for domain gains in different species after species divergence. That is why we looked at the characteristics of gained domains in representative proteins of each species separately. We found that gain of multiple terminal novel exons is a dominant mechanism for domain gains in human, mouse and frog (these gains accounted for 34, 50 and 56%, respectively, of all gains with representative protein in these species); in fruit fly the dominant category was extension of an exon at the carboxyl terminus (29% of domain gains); and in zebrafish it was a mixture of the two (35% of gains were novel terminal domains and 20% carboxyl terminus exon extensions). For rat and chicken we had too few domain gains to draw conclusions.

Recent segmental duplications in the human genome are a possible source of new genetic material [45] and their role in the evolution of primate and human specific traits has been debated [46]. Hence, we investigated whether recent domain gains in the human lineage could be related to the reported segmental duplications. We found two domain gains that were best explained by recent segmental duplications and subsequent joining of two genes (Additional file 8c,d). Both of these gains occurred at the protein termini after divergence of primates. The mechanism of their evolution is the same as in the case of the *UEVLD *gene: joining of exons from adjacent genes after gene duplication. For these two examples, however, there is also evidence of a likely connection between recent genomic duplication and domain gain. However, it is necessary to be cautious when assessing the possible role of the protein products of these genes. For both examples, there is only transcript evidence and some of the transcript products of these genes appear to have a structure that would lead to them being targeted by nonsense-mediated decay (NMD) [47]. Sometimes it is possible for a transcript to avoid an NMD signal and in this case these examples would be of high interest as possible sources of novel function. A possible mechanism for the creation of these proteins is illustrated in Additional file 8c,d. In the case that these transcripts are silenced by NMD, these genes are still interesting examples from a theoretical point of view as they directly illustrate the mechanism of how gene evolution can work. Initially, part of a gene sequence is duplicated and recombined with another gene; if juxtaposed exons are in frame, a joint transcript can be created and through NMD deleterious variants can be silenced at the transcript level while allowing at the same time introduction of novel mutations that can be tested by natural selection.

### The dominant mechanism for domain gains relies on gene duplications

One advantage of using TreeFam phylogenies is the ability to distinguish between gene evolution that follows gene duplication and gene evolution that follows speciation. When comparing the observed versus expected frequency of duplication and speciation events after which domain gain occurred, we found that domains were gained 2.7 times more frequently after gene duplication compared to after speciation (if calculations were performed using branch lengths) and 4.5 times more frequently when numbers of nodes were compared (see Additional file 7 for details). This shows that duplication of not only the 'donor gene' but also of the 'recipient gene' assisted domain gains. Taken together, in 80% of our domain gain events, duplication of either the ancestral protein or donor protein has been involved. Moreover, when two genes were fused together then the assignment of 'donor' and 'recipient' genes depends solely on whose phylogeny we are looking at.

**Figure 5:**
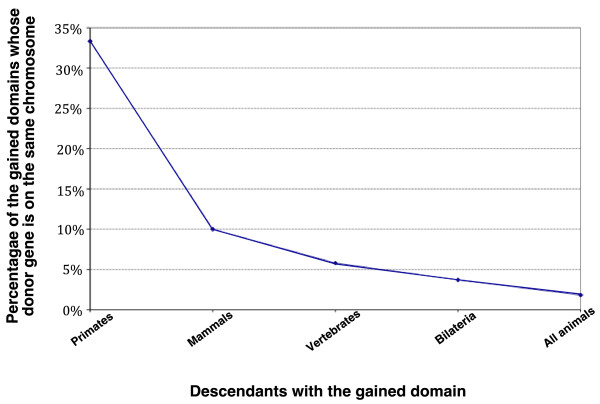
**Chromosomal position of the 'donor gene' and the relative age of the gain event**. The graph shows the fraction of events for which the 'donor gene' of the gained domain is identified, and is on the same chromosome as the gene with the gained domain, with respect to the relative age of the gain event. The gain events were divided into five groups according to the expected age of the event as judged by the TreeFam phylogeny. The x-axis shows the evolutionary group in the human lineage to which descendants of the gain event belong, and the y-axis shows the percentage of gain events in each evolutionary group for which both of the conditions were valid: we were able to find the donor gene and the donor gene was on the same chromosome as the gene with the gained domain. This was true for 3 out of 9 gain events in primates, 2 out of 20 in mammals, 7 out of 121 in vertebrates, 1 out of 27 in Bilateria and 1 out of 55 in all animals. Estimated divergence times (in millions of years ago (mya), as taken from Ponting [80]) are: 25 mya for primates, 166 mya for mammals, 416 mya for vertebrates and 700 mya for all animals (we were not able to estimate the divergence time for Coelomata).

When it is possible to find the origin of the duplicated domain, the overall trend is that the younger the gain is, the more likely it is that the 'donor gene' is on the same chromosome as the 'recipient gene' (Figure 5). NAHR creates duplicates more frequently than IR does [48,49], creates them preferentially on the same chromosome [48], and provides ground for gene rearrangements. Therefore, it is possible that NAHR assisted domain gains, and in particular preceded joining of exons from adjacent genes. We do not exclude IR as a possible causative mechanism but NAHR seems more likely given the bias in chromosome locations of domain duplicates and the reliance of the gain mechanism on gene duplication (further discussed in Additional file 7).

### Functional implications of domain gain events

It has been proposed that the novel combinations of preexisting domains had a major role in the evolution of protein networks and more complex cellular activities [5,50]. In agreement with this, we found that the most frequently gained protein domains in the human lineage - domains independently gained five or more times in our set - are all involved in signaling or regulatory functions; the Ankyrin repeat (gained six times) and SAM domain (gained five times) are commonly involved in protein-protein interactions, and the Src homology-3 and PH domain-like superfamily (both gained six times) frequently have a role in signaling pathways. Furthermore, we used DAVID [51] to investigate if human representative transcripts (from Additional file 5) were enriched in any Gene Ontology terms. Significantly enriched Gene Ontology terms are listed in Additional file 10 and are, in general, involved in signal transduction; among the significant terms are 'adherens junction', 'protein modification process' and 'regulation of signal transduction'. This further supports the role of novel domain combinations in the evolution of more complex regulatory functions.

## Discussion

Creation of novel genes is assumed to play a crucial role in the evolution of complexity. Previous studies have put considerable effort into identifying gene gain and loss events during animal evolution, as well as analyzing functional and expression characteristics of these genes [52-56]. In this study, our aim was to investigate functionally relevant changes of individual proteins. Implications of observed domain gains on the evolution of more complex animal traits are highlighted by the frequent regulatory function of the gained domains in the human lineage. Shuffling of regulatory domains has already been proposed as an important driving force in the evolution of animal complexity [5,50], and an increase in the number of regulatory domains in the proteome has been directly related to the increase of organismal complexity [57].

The relative frequencies of domain gain and loss events are not known and most probably are not universal for different domains and organisms. Hence, different approaches have been undertaken to address this issue. Several previous studies have assumed that the frequencies of gain and loss events are equal and have identified domain gains and losses by applying maximum parsimony [58-61]. Other studies have assumed that domain loss is slightly more likely than domain gain [62] or that the difference in the frequency of gains and losses is very significant and hence have suggested Dollo parsimony - which allows a maximum of one gain per tree - for identifying domain gains [63,64]. In genomes in which proteins often have several domains, one can expect that the mechanisms that cause domain loss are more frequently at play than the mechanisms that cause domain gain. In particular, exclusion of domains could be an effective means for subfunctionalization after gene duplication. For instance, mutations that introduce a novel stop codon or that cause exon skipping during alternative splicing can easily shorten the protein. Hence, in the studies of multidomain animal proteins, one should be careful about applying simple maximum parsimony since it can happen that the number of domain gains is falsely overestimated - when in fact multiple losses have occurred. In particular, in this study, it was crucial to identify high-confidence cases of domain gains. Our approach to do this was to be very strict about calling domain gains: we applied the weighted parsimony algorithm assuming that it is two times more likely for a protein to lose a domain than to gain a new one; additionally, we classified an event as a domain gain only if a single gain of a particular domain was reported in a tree, which is the rationale of the Dollo parsimony. If we had applied Dollo parsimony only we would not have been able to distinguish between eventual multiple gains of the same domain, and this approach excluded such dubious cases. This strategy appeared to remove a number of possible false domain gains as judged by inspection of the results.

Present domain combinations are shaped by the causative molecular mutation mechanisms followed by natural selection. Here we address the question of what mechanisms have been, and possibly still are, creating novel, more complex animal domain architectures and hence new functional arrangements. Our data suggest that the dominant mechanism has been gene fusion through joining of exons from adjacent genes and that the process of domain gain has strongly relied on gene duplication. In this study we find novel examples that directly illustrate this mechanism; after duplication, exons that encode one or more domains are joined with exons from another adjacent gene. The examples are interesting both from the point of view of the evolution of protein diversity and as examples for novel gene creation during animal evolution. It is possible that recombination created novel introns and directly joined exons from two adjacent genes, but it is more likely that recombination only juxtaposed novel exon combinations, allowing alternative splicing to create novel splice variants. There are indications that NAHR could have caused the initial duplications and rearrangements. The implications for the role of NAHR in animal evolution in general are particularly interesting since this mechanism is still primarily associated with more recent mutations in the human genome, as well as primate genomes in general, such as structural variations in the human population and disease development [46,65,66]. It has recently been proposed, however, that the fork stalling and template switching (FoSTeS) mechanism [67] could have also had a role in genome and single-gene evolution. This is a replicative mechanism that relies on microhomology regions and seems to provide a better explanation for complex germline rearrangements - but also for some tandem duplications in the genome - than NAHR and IR [68]. Hence, the exact relative contributions of different recombination mechanisms are still to be determined. However, this might be hampered by sequence divergence after domain gain events, which have occurred millions of years ago.

In this work, we also address exonization of previously non-coding sequences as a mechanism for gain of novel domains. We observe that domains that are gained as exon extensions are preferentially disordered (Figure 3). This suggests that exonization of previously non-coding sequences could explain some cases of evolution of disordered protein segments in animal proteins. Disordered segments in higher eukaryotes are linked with important signaling and regulatory functions [69,70] and inclusion of these sequences into proteins, together with creation of novel domain combinations, could have added to the emergence of complexity in higher eukaryotes. An illustration from the literature for the significance of inclusion of novel disordered segments into proteins is the evolution of NMDA (N-methyl-D-aspartic acid) receptors. These receptors display a vertebrate-specific elongation at the carboxyl terminus. Gained protein regions are disordered and govern novel protein interactions, and it is believed that this might have contributed to evolution and organization of postsynaptic signaling complexes in vertebrates [71]. Moreover, our data suggest that there is a bias for exon extensions to preferentially occur at the carboxyl terminus (Figure 2), which is in agreement with the assumption that some of these domain gains occurred through exon extension since extension of exons at the amino terminus or in the middle of proteins can introduce frame shifts and hence can be selected against. However, Pfam families that are classified as exon extensions are also likely to be shorter, so it is possible that this introduces some bias because shorter families are less likely to be domains with defined structures. Moreover, an important caveat is that only a systematic study can confirm domain gain by this mechanism; apparently non-coding sequences that are homologous to gained domains might just lack transcript and protein evidence in the less studied species, resulting in a domain assignment being missed.

Finally, it is important to note that even though we have attempted to draw conclusions about dominant mechanisms for evolution of animal genes, it is possible that contributions by different mechanisms will differ between different species. Percentages of active retrotransposons and rates of chromosomal rearrangements and intergenic splicing are different in different genomes, as are the selection forces that depend on population size and that decide on how well tolerated intermediate stages in gene evolution are. Therefore, it is possible that we will find out that some mechanisms are more relevant in some species than they are in others. This is illustrated by differences in characteristics of gained domains in vertebrates and *Drosophila*. The dominant mechanism in *Drosophila *seems to be extension of exons at the carboxyl terminus. Additionally, even though the majority of gain events are represented by human proteins, different mechanisms could have dominated at different evolutionary time points in the human lineage. For example, LINE-1 retrotransposons are abundant in mammals but not in other animals [72], and whole genome duplication that occurred after the divergence of vertebrates [73] could have preferred recombination between gene duplicates at that point in time.

Retroposition and recombination-assisted intronic insertions, in contrast to previous expectations, appear to be minor contributors to domain gains. Therefore, it is possible that the role of intronic insertions had been overestimated previously. It will be interesting to see if the observed excess of symmetrical intron phases around exons coding for domains [25] is due to exon shuffling or to some other mechanism, such as selective pressure from alternative splicing [74]. In conclusion, our work provides evidence for the importance of gene duplication followed by adjacent gene joining in creating genes with novel domain combinations. The role of duplicated genes in donating domains to adjacent proteins is a potentially important, and powerful, mechanism for neofunctionalization of genes.

## Conclusions

We report here a large-scale analysis of the mechanisms that have caused domain gains in animals and describe several novel examples that illustrate gene evolution in the human lineage. Our study suggests that joining of exons from adjacent genes has played a crucial role in the evolution of novel human genes. Moreover, it indicates a strong link between gene duplication and the invention of novel domain combinations, thus implying a powerful means for the fast evolution of novel function after gene duplication.

## Materials and methods

Here we describe the procedures used to identify a set of high confidence protein domain gains and the subsequent analyses of this set. This flow is illustrated in Additional file 2a,b.

### Assignment of domains to proteins with refinement

We assigned Pfam domains (release 23.0) to all protein products of genes in the TreeFam database (release 6.0) using the Pfam_scan.pl software. Since domains in the same Pfam clan are evolutionarily related, we replaced domain identifiers with clan identifiers where applicable. Domain prediction methods can both fail to predict *bona fide *domains as well as make false predictions, which look like domain losses and gains, respectively. We applied a refinement process to address this issue. We firstly removed the likely false positive fragmentary domain assignments (that is, domains that were called on only a single sequence in the family, with an E-value > 10^-6 ^and only 30% or less of the domain's Pfam model covered). Additionally, when some sequences lacked a domain that other family members had, we used Wu-blastp to search that sequence against the domain sequences found in other members of the family. When a significant match was found (E-value < 10^-4 ^and at least 60% of a domain sequence present, or alternatively an E-value < 10^-7 ^and 40% or more of a domain sequence present, or only E-value < 10^-10 ^and any length of the matched sequences) we added domain assignments to those sequences.

### Exclusion of possible false domain gain calls

Domain refinements added Pfam domains to proteins that shared significant similarity with domain sequence but were not recognized by searching with the Pfam hidden Markov model library. However, apart from these clear cases of a lack of domain annotation, there are also cases where proteins share only moderate similarity with domain sequence and it is difficult to say whether a domain should be annotated to these proteins as well. To avoid false calls of domain gains, we excluded domain gain events where sequences in the same gene family shared a similarity with the gained domain but were not annotated with that domain. We chose a strict threshold and excluded all gain events where a domain sequence had 16% or more identical amino acids aligned to any sequence in the same TreeFam family that lacked the gained domain. This further reduces the chances of erroneously calling domain gains due to a lack of sensitivity of some Pfam hidden Markov models.

### Parsing trees

To identify the branch points in the phylogenetic trees at which new domains were gained, we used the TreeFam API [28]. In TreeFam families each gene is represented with a single transcript. However, to be able to claim that a gene has gained a domain it was necessary to take into account protein domains present in all splice variants of the genes in the TreeFam families. We applied the weighted parsimony algorithm [75] on the TreeFam phylogenies, with the cost for a domain gain of 2 and the cost for a domain loss of 1. Because gains are more costly, the ones we see are more likely to be correct. We then took into account those reported gain events that occurred only once in a tree - which is the rationale of the Dollo parsimony [76]. We applied this method to the 17,050 TreeFam clean trees, that is, trees containing genes from completely sequenced animal genomes. We considered the gained events to be the ones that were in concordance with both algorithms - 4,362 gained domains. We then excluded from the analysis those gains that appeared only on the leaf nodes of the trees - that is, that had only one sequence with the gained domain - and were left with 1,372 domains gained on internal nodes of the tree. Next, we aimed to chose a representative transcript for each gain event, and the conditions for that were the following: the transcript had to be present in the TreeFam tree (the gains were also reported when the gene gained another alternative transcript, not only when the TreeFam transcript was extended with a new sequence); the transcript had to have a gained domain on the encoded protein predicted by the Pfam software; and the representative transcript had to belong to one of the species *D. melanogaster *(fruit fly), *Xenopus tropicalis *(frog), *Danio rerio *(zebrafish), *Gallus gallus *(chicken), *Mus musculus *(mouse), *Rattus norvegicus *(rat) or *Homo sapiens *(human) - that is, to a species whose genome is of a good quality. This left us with 653 gained domains that had representative transcripts that fulfilled all conditions. Since each representative sequence was chosen from the descendant with the genome of best quality for all gains in the human lineage, we chose representative human transcripts (proteins). Exclusion of leaf gains and selection of representative transcripts from better quality genomes were necessary to ensure that our gain events were not due to gene annotation errors. We then excluded all instances where the sequences from the same family that lacked the gained domain were found to have diagnostic motifs for that domain, as recognized by profile comparer [77], or to have an amino acid stretch similar to one in the gained domain (16% or more identical amino acids). This left us with 378 gained domains. Some of these domains appeared to be gained together so the total number of domain gain events was 349. Finally, we excluded from the analysis the gain events for which a representative transcript was no longer in the Ensembl database, release 50 (3 cases) or for which protein sequence alignment downloaded from the TreeFam database did not clearly support domain gain (13 cases) and those cases that we believed were the consequence of inconsistencies in gene annotation (3 cases; Additional file 7). This left us with a final total of 330 high-confidence domain gain events for further analysis (Additional file 5). In addition, we created a medium-confidence set of domain gain events. For this, we only asked the gain to occur in at least one genome of better quality. However, this also increased the rate of false calls of domain gains. This left us with 849 gained domains. The flow to obtain this set of gains is shown in Additional file 2a.

### Intron-exon structures of genes

We used the TreeFam table map with gene structures to project the intron-exon boundaries and intron phases on the representative protein sequences for each domain gain event. In order to establish whether the gained protein domains were part of completely new exons or extensions of already pre-existing exons, we downloaded protein sequence alignments for each TreeFam family with a gained domain from the TreeFam website. Since it is unlikely that the gained sequence would exactly correlate with domain boundaries, we examined the similarity in regions close to exon boundaries. We considered that a domain was inserted into an existing exon if the region in the same exon close to the exon border shared partial similarity with an exon from those sequences in the same family that lacked the domain. We considered that the domain was gained within an existing exon when the boundary region of the exon - first or last third of the sequence outside of the domain - had 30% or more identical residues to one of the sequences without the inserted domain. We required that this 'boundary' region was at least seven amino acids long. However, because of this criterion that only a short stretch of sequence similarity is enough to claim that a gained domain is coded by an extended ancestral exon, the number of extended exons is likely to be an overestimate.

### Positions of gained domains

When a new domain was encoded by a first or last coding exon, the gain was called as an amino- or carboxy-terminal gain, respectively. In addition, when an inserted domain was not coded by the terminal exons, we checked whether additional exons towards the termini were gained together with the ones coding for the gained domain. If there was no significant similarity between these exons and the ones in the sequences without the gained domain, the exons were called novel and the gain still called terminal. Conditions for calling an exon as novel were: 85% or more novel amino acids in an exon (that is, residues unaligned with amino acids in the sequences without the domain); or less then 10% identity with any of the sequences without the domain. For short exons coding for 20 amino acids or less, we changed this requirement to less than 40% identity. All other domain gains were classified as middle.

It is important to note that examining the sequences that surround the gained domains, when classifying the gains according to their relative position and as exonic or intronic, also helps to overcome the issue of imperfect domain boundary assignments, which could bias classification of gained domains.

### Genomic origin of the inserted domain

For all domain gain events that have a human descendant, the gained domain sequence from a representative protein was searched against the rest of the human proteome. For this we used Wu-blastp. The best significant hit that was not in one of the gene's paralogues was considered to be a potential donor of the gained domain. A set of paralogues for each gene was composed of other human genes from the same TreeFam family and Ensembl paralogues for that gene. The condition for a significant hit was an E-value < 10^-4 ^with 60% or more of the domain sequence aligned. We visually examined the structures of the genes with gained domains and of their best hits using Ensembl (release 50) and the Belvu viewer [78].

We used the segmental duplication coordinates from the Segmental Duplication Database [79]. We investigated whether any segment from the database overlapped with any of the representative genes with a domain gain, and if so, whether the other copy of that segmental duplication was placed on the gene that was a potential donor of that domain. We also checked whether the other copy overlapped with any of the paralogs of the representative gene.

## Abbreviations

IR: illegitimate recombination; NAHR: non-allelic homologous recombination; NMD: nonsense mediated decay.

## Authors' contributions

MB participated in design of the study, carried out analyses and drafted the manuscript. AF analyzed gene structures of selected examples. AB conceived of the study and participated in design of the study and writing of the manuscript. All authors read and approved the final manuscript.

## Supplementary Material

Additional file 1 **Further discussion of different types of domain gain events as classified in Figure **2. Click here for file

Additional file 2 **Flowchart of (a) methods and (b) analysis for the set of high-confidence domain gain events and for the set of medium-confidence domain gains**. The numbers of gained domains we were left with after each filtering step are noted in (a). In some cases more domains were gained at the same time; hence, the number of gain events that we looked at for the high-confidence domain gains differs from the number of gained domains. Click here for file

Additional file 3 **Distribution of domain gain events according to the position of the domain insertion and the number of exons gained in the set of high-confidence domain gains and the set of medium-confidence domain gains**. **(a) **The distribution of characteristics of domains from the high-confidence set of domain gains is identical to that in Figure 2. **(b) **The distribution of characteristics of domains from the set of medium-confidence domain gains. There are in total 330 high-confidence domain gain events and 849 medium-confidence domain gains (of which 19 gains have ambiguous position and are not shown in the graph). The flowchart in Additional file 2a shows the procedure for creation of these two sets of domain gains. The distribution of domain gains in the medium-confidence set (b) is similar to that in the set of high-confidence domain gains; the main difference is that the number of middle domain gains is increased. We believe that this is largely due to false domain gain calls caused by some proteins in the TreeFam families missing the Pfam annotations for domains that are actually present in these proteins. Click here for file

Additional file 4 **Analysis of supporting evidence for the representative transcripts for domain gain events**. Click here for file

Additional file 5 **A table listing high-confidence domain gain events**. Click here for file

Additional file 6 **Analysis of evidence for retroposition and middle insertions by intronic recombination as mechanisms for domain gain**. Click here for file

Additional file 7 **Fusion of adjacent genes and NAHR as a mechanism that preceded gene fusions**. Discussion of evidence for NAHR as a mechanism that frequently assisted domain gains. Click here for file

Additional file 8 **Examples of domain gains by joining of exons from adjacent genes**. **(a) **TreeFam family TF323983 contains Cadherin EGF LAG seven-pass G-type receptor (*CESLR*) precursor genes. One branch of the family, containing vertebrate genes, has gained the Sulfate transport and STAS domains in addition to the ancestral cadherin, EGF and other extracellular domains. The gain occurred after the other vertebrates diverged from fish and homologues without the gained domains are present in all animals. A representative for the gain is the transcript *CELSR3-207 *(*ENST00000383733*) and its 3' end is shown on the left-hand side (the whole transcript is too long to be clearly presented). On the right-hand side is shown a gene that is the plausible donor of these domains. Namely, the gene *SLC26A4 *(*ENSG00000091137*) contains both domains, and its STAS domain is 31% identical to that in the *CELSR3 *gene. In addition, the alignment with the zebrafish genome is shown below the *CELSR3-207 *transcript. The yellow arrows represent the alignment with chromosome 8 in zebrafish, and pink arrows that with chromosome 6 (information taken from the USCS browser). The alignment with the fish genome shows that the synteny is broken exactly in the region where the new domain is gained. Therefore, the plausible scenario for domain gain involves gene duplication, recombination and joining of newly adjacent exons. **(b) **Another example of a domain gain after gene duplication and exon joining. Family TF334740 in the TreeFam database contains genes that code for the Rho-guanine nucleotide exchange factor (RhoGEF). However, the RhoGEF domain was not present in the ancestral protein but was inserted later on together with the C1_1 domain when mammals diverged from other vertebrates (TreeFam release 6.0 that we used in the analysis had chicken, fish and frog genes without the gained domains). The representative transcript for the gain event is *AC093283.3-201 *(*ENST00000296794*). The gene *ARHGEF18 *(*ENSG00000104880*) has both of these domains, and the two RhoGEF domains between the genes are 52% identical. Hence, ARHGEF18 is a plausible donor for this gain event. Again, the mechanism for the gain of these domains most likely involves gene duplication and exon joining. **(c) **An example of a domain gain after segmental duplication and exon joining. TreeFam family TF351422 contains only primate genes, and after a gene duplication event one branch of the family has gained the PTEN_C2 domain. A representative transcript for this gain is *AL354798.13-202 *(*ENST00000381866*). A few segmental duplications span across the gene *AL354798.13 *and one of them covers only the ancestral portion of the gene - without the gained domain. The pair of that segmental duplication is on the gene's paralogue that has not gained the domain, the gene *AP000365.1 *(*ENSG00000206249*). Hence, a possible scenario is that a recent duplication of a paralog gene has changed its genetic environment and brought it into proximity of the PTEN_C2 domain, which subsequently became part of the gene. **(d) **Another example of a gain of a domain-coding region by segmental duplication followed by exon joining. A branch with primate genes in the TF340491 family of vertebrate proteins that contain the KRAB domain has gained the additional HATPase_c domain. The representative transcript is the human *PMS2L3-202 *(*ENST00000275580*). The HATPase_c domain exists in the gene *PMS2 *(*ENSG00000122512*) and on the protein level the gained domain is 98% identical to the sequence in the protein product of PMS2's transcript, *PMS2-001*. A segmental duplication spans across the gained sequence in the transcript *PMS2L3-202 *and is a pair of the segmental duplication that covers the same domain in the gene *PMS2*. The pair of segmental duplication regions are presented as grey boxes and are connected with arrows. Therefore, the mechanism underlying this gain appears to be a segmental duplication of the sequence belonging to *PMS2 *after which the copy next to *PMS2L3-202*'s ancestor was joined with it. An important caveat is that PMS2L3-202 has a structure that can be targeted by NMD. Click here for file

Additional file 9 **A table that lists domains that are classified as being gained by insertion of new exons(s) into the introns of ancestral genes**. Click here for file

Additional file 10 **A table listing significant Gene Ontology terms for human genes that have been extended with a new protein domain during evolution**. Click here for file
