# Supplementary material for: Quantifying the mechanisms of domain gain in animal proteins
Source: Genome Biol. 2010 Jul 15;11(7):R74. doi: 10.1186/gb-2010-11-7-r74 (PMC2926785; doi:10.1186/gb-2010-11-7-r74)
Supplement: Additional file 1 — Further discussion of different types of domain gain events as classified in Figure 2. [file gb-2010-11-7-r74-S1.DOC]

**Further discussion of different types of gain events as classified in Figure 2**

In the main text we discuss the gains of multiple novel terminal exons as these events make up 32% of all domain gains and there is only one likely mechanism that could have caused these gains. Terminal gains of domains coded by a single novel exon can be explained either by the joining of exons from adjacent genes or with other mechanisms such as retroposition. The former mechanism is more likely since, together with the novel exon that codes for the gained domain, extra exons, that do not code for the gained domain, have frequently been gained (in at least 42% events, or 18 of total 42 cases). Also further inspection of the candidate gains in the human lineage did not find LINE elements that preceded a gained or ‘donor’ domain and hence did not lend support for retroposition as a causative mechanism (Additional file 6). With regard to other categories of domain gain events in Figure 2, because of the strict criteria used to call a gained domain terminal and coded by novel exons, a number of exon extensions and middle gains are probably misclassified terminal gains and gains of novel exons. Possible mechanisms for the real exonic extensions are gene recombination inside exon regions, deletion of sequences between exons of two adjacent genes or exon extension through exonisation of previously non-coding regions. We were particularly intrigued by C-terminal single exon extensions because there appeared to be a bias of these with respect to the position in a protein in Figure 2. We looked at alignments for these gains in the human lineage and found four convincing examples for true exon extensions. None of these had a potential origin identified in the human proteome. Further inspection of these domains showed that they have actually occurred at that point in the evolution for the first time and the possible mechanism for inclusion of these novel domains was reading through the stop signal and exonisation of previously non-coding sequences (for the gains in primates and mammals alignments at UCSC genome browser [32] show similarity of the gained domains with non-coding regions in the genomes of non-primates and non-mammals, respectively). These examples are: (1) Gain of a proline rich Pfam family PF04680 in primates – in the TreeFam family TF331377, (2) gain of a selenoprotein P C-terminal Pfam family PF04593 in mammals – in the TreeFam family TF333425, and gain of the families: (3) connexin 50 C-terminal - PF03509 and (4) the Kv2 voltage gated K^+^ channel - PF03521 in vertebrates – in the TreeFam families TF329606 and TF313103, respectively. Representative transcripts for these gains can be found in Table S1. It is noteworthy that none of these Pfam families has a solved structure and it is possible that they are not true structurally independent protein domains. Even so, their sequences are conserved in the organisms in which these Pfam families are present (it was possible to recognize these domains in the sequence). This implies that they could be functionally relevant. We also had a closer look at domain gains after primate divergence and have found that two of domain gain events are actually gains of transposon (PF02023 and CL0219 the TF328297 family) and retroviral (CL0074 in the TF331083 family) domains. Gains of domains from mobile genetic elements can also be relevant for the evolution of protein function [21].

To calculate the statistical significance of the observation that gains at protein termini are preferred over the gains in the middle of a protein, we looked at domain architectures of proteins with domain gains. In total, there were 330 domain gain events in which 359 domains were gained. We looked at each domain separately, so when the gain occurred at termini only one domain was assigned as a terminal domain, and all others as middle domains. Out of the gained domains 301 were observed at protein termini and 58 in the middle of proteins. Note that domain which is classified as a middle gain in our study can still be at termini in protein domain architecture. Out of all other domains in these proteins 279 were at protein termini, and 181 in the middle of proteins. Thus, terminal domains were significantly overrepresented among the gained domains (P-value < 7.7 x10^-13^, Chi-square test).
