# Supplementary material for: Quantifying the mechanisms of domain gain in animal proteins
Source: Genome Biol. 2010 Jul 15;11(7):R74. doi: 10.1186/gb-2010-11-7-r74 (PMC2926785; doi:10.1186/gb-2010-11-7-r74)
Supplement: Additional file 4 — Analysis of supporting evidence for the representative transcripts for domain gain events. [file gb-2010-11-7-r74-S4.DOC]

**Supporting evidence for the representative transcripts**

We based this work on the Ensembl gene and transcript predictions. However, Ensembl predictions rely on the supporting transcriptome and proteome evidence which is still incomplete. Mistakes in the transcript models can cause false domain gain calls for two reasons: firstly, a transcript that has apparently gained a domain coding sequence can actually exist as two separate transcripts that are falsely annotated as one longer transcript, and secondly, if a domain gain is reported in the genomes with better quality annotations it could be that in the genomes of lower quality the domain is missing only due to incomplete annotation.

To investigate the possible extent of errors introduced by the first type of annotation errors, we checked if there was available supporting evidence for the transcripts that were representatives for domain gain events. We retrieved supporting evidence on the transcript level by using the Ensembl API and checked individual human and mouse representatives without the supporting evidence through the Ensembl website. We found that there was known mRNA supporting the transcript structure in 226 out of 232 human representative gain events and that there were 4 additional cases where evidence was on the exon level. Therefore, 99% (230 of 232) of human representatives have valid supporting evidence. For mouse, the percentage is a bit lower; there is evidence on the transcript level for 14 out of 18 representative gain cases, and two other transcripts are supported on the exon level. Hence, supporting evidence exists for 89% of the gain events (16 of 18) with mouse representative transcript. For other organisms we took only automatically retrieved transcript evidence into account and we found that in rat there was supporting evidence for 60% (3 of 5) of the events, in chicken and zebrafish for 25% (1 of 4 and 5 of 20 events, respectively), and for frog and fruit fly none of the representative transcripts had available supporting evidence (there were 9 and 43 representative transcripts in frog and fruit fly respectively). Therefore, we feel confident about the transcripts with gained domains in human and mouse, but are more cautious about representative transcripts with the gained domain coding sequences in other organisms.

We addressed the level of possible false domain gain calls due to the second type of annotation errors on a smaller set of domain gains which represented a set of gain calls likely to be affected by this error. Namely, domain gains that occurred in the human lineage after the divergence of vertebrates (121 reported domain gain events) can have on one side well studied genomes as human and mouse and on the other side, as an outgroup, lower quality annotation genome of *C. intestinalis*. For 49 of these gain events the TreeFam family with the reported domain gain also contained orthologous genes in C. *intestinalis* without that domain. We took sequences of C. *intestinalis* orthologous together with the 5kb of sequence upstream and downstream of them and performed tBLASTn [33] to test whether the missing domains were present but only lacked annotation. We found that in four cases at least one of the domains reported to be gained in vertebrates is present in the neighbourhood of C. *intestinalis* orthologous (P-value < 0.1, tBLASTn). However, for two of these cases gene annotation is of very good quality, and the predicted UTR signals and proximity to their neighbouring genes do not support hypothesis that the ‘missing domains’ should be added to these genes. Therefore, we estimate that 4% (2 of 49) of the apparently gained domains could be reported due to errors in gene annotations. However, since these domains are found only in vertebrate genes in the corresponding TreeFam families, these might still be the cases of domain gain but only the time points of the gain events could be before the divergence of *C. intestinalis* from vertebrates. Domains found next to the C. *intestinalis* orthologues, which are possibly missed by incomplete gene annotations were: PF03160 next to ENSCING00000003141 which was gained in TF105392 together with CL0159, and CL0084 next to ENSCING00000005839 which was gained in TF329720 together with PF00533. The other two domains which are found next to *C. intestinalis* genes which do have good quality annotation are PF01403 next to ENSCING00000006805 which was gained in TF317402, and a domain PF00014 next to ENSCING00000011322 which was gained in TF331207.
