# Supplementary material for: Quantifying the mechanisms of domain gain in animal proteins
Source: Genome Biol. 2010 Jul 15;11(7):R74. doi: 10.1186/gb-2010-11-7-r74 (PMC2926785; doi:10.1186/gb-2010-11-7-r74)
Supplement: Additional file 5 — A table listing high-confidence domain gain events. [file gb-2010-11-7-r74-S5.DOC]

**Table S1:** **High confidence** **domain gain events.** Information about descendants of the gain event is shown only for the gains in the human lineage.

| **TreeFam family** | **Pfam domain** | **Representative transcript** | **Descendants** |
| --- | --- | --- | --- |
|  |  |  |  |
| TF340491 | PF02518 | ENST00000275580 | Primates |
| TF331377 | PF04680 | ENST00000290291 | Primates |
| TF352220 | PF05033,PF00856 | ENST00000307483 | Primates |
| TF331083 | CL0074 | ENST00000338965 | Primates |
| TF342157 | PF04698 | ENST00000354668 | Primates |
| TF328297 | CL0219,PF02023 | ENST00000357581 | Primates |
| TF340395 | CL0159 | ENST00000359050 | Primates |
| TF314793 | PF00271,CL0008 | ENST00000370424 | Primates |
| TF351422 | PF10409 | ENST00000381866 | Primates |
| TF105356 | CL0023 | ENST00000194097 | Mammals |
| TF335271 | CL0041 | ENST00000254691 | Mammals |
| TF328011 | PF02023 | ENST00000259883 | Mammals |
| TF337552 | PF00096 | ENST00000262637 | Mammals |
| TF328424 | PF05386 | ENST00000262715 | Mammals |
| TF337951 | PF00147 | ENST00000301455 | Mammals |
| TF300253 | PF03002 | ENST00000320498 | Mammals |
| TF350810 | PF01352 | ENST00000338637 | Mammals |
| TF338854 | PF01352 | ENST00000344099 | Mammals |
| TF331962 | PF00612 | ENST00000366709 | Mammals |
| TF338165 | PF04711 | ENST00000367990 | Mammals |
| TF336000 | PF08065 | ENST00000368654 | Mammals |
| TF330114 | CL0175 | ENST00000373330 | Mammals |
| TF333425 | PF04593 | ENST00000388827 | Mammals |
| TF325887 | PF10522 | ENST00000394516 | Mammals |
| TF105660 | PF08062 | ENST00000399466 | Mammals |
| TF330855 | PF03523 | ENST00000262101 | Mammals |
| TF334740 | CL0006,PF00621 | ENST00000296794 | Mammals |
| TF329807 | PF06049 | ENST00000367797 | Mammals |
| TF324004 | PF02008 | ENST00000373644 | Mammals |
| TF317779 | PF09307 | ENST00000009530 | Vertebrates |
| TF326567 | CL0003 | ENST00000046794 | Vertebrates |
| TF325130 | PF00023 | ENST00000160373 | Vertebrates |
| TF106374 | CL0172 | ENST00000199447 | Vertebrates |
| TF105392 | CL0159,PF03160 | ENST00000200181 | Vertebrates |
| TF325426 | PF00632 | ENST00000206595 | Vertebrates |
| TF319848 | PF01033 | ENST00000229003 | Vertebrates |
| TF106352 | PF00023 | ENST00000230792 | Vertebrates |
| TF313285 | PF01759,PF01821 | ENST00000245907 | Vertebrates |
| TF329176 | CL0081 | ENST00000249910 | Vertebrates |
| TF330078 | PF10393 | ENST00000255132 | Vertebrates |
| TF320327 | PF04812 | ENST00000257555 | Vertebrates |
| TF320327 | PF04813 | ENST00000257555 | Vertebrates |
| TF330114 | PF05485 | ENST00000260045 | Vertebrates |
| TF331062 | CL0072 | ENST00000260283 | Vertebrates |
| TF313938 | CL0154 | ENST00000260983 | Vertebrates |
| TF316484 | PF01463,CL0022,PF01822 | ENST00000262304 | Vertebrates |
| TF316148 | PF03815 | ENST00000262424 | Vertebrates |
| TF312824 | PF00612 | ENST00000262457 | Vertebrates |
| TF316113 | CL0003 | ENST00000262878 | Vertebrates |
| TF317402 | CL0159 | ENST00000263798 | Vertebrates |
| TF317511 | PF00017 | ENST00000263915 | Vertebrates |
| TF331945 | PF07525 | ENST00000264607 | Vertebrates |
| TF316876 | PF00093 | ENST00000264895 | Vertebrates |
| TF316876 | PF03160 | ENST00000264895 | Vertebrates |
| TF324610 | PF02732 | ENST00000267430 | Vertebrates |
| TF351678 | PF01392 | ENST00000273857 | Vertebrates |
| TF314731 | PF10565 | ENST00000279593 | Vertebrates |
| TF313240 | PF10606 | ENST00000282753 | Vertebrates |
| TF316380 | CL0011 | ENST00000283296 | Vertebrates |
| TF316380 | PF01390 | ENST00000283296 | Vertebrates |
| TF351678 | CL0202 | ENST00000284885 | Vertebrates |
| TF329158 | CL0023 | ENST00000285928 | Vertebrates |
| TF314232 | PF00569,CL0220 | ENST00000288642 | Vertebrates |
| TF316484 | PF02010 | ENST00000289672 | Vertebrates |
| TF332664 | PF07776 | ENST00000289816 | Vertebrates |
| TF313285 | CL0005 | ENST00000291440 | Vertebrates |
| TF336193 | PF01342,PF03172 | ENST00000291582 | Vertebrates |
| TF323966 | CL0214 | ENST00000294383 | Vertebrates |
| TF317402 | PF01403 | ENST00000296474 | Vertebrates |
| TF329295 | CL0124 | ENST00000296498 | Vertebrates |
| TF329059 | CL0001 | ENST00000296575 | Vertebrates |
| TF331157 | CL0041 | ENST00000297350 | Vertebrates |
| TF312852 | CL0219 | ENST00000298139 | Vertebrates |
| TF323475 | CL0003 | ENST00000298229 | Vertebrates |
| TF323480 | CL0005 | ENST00000302495 | Vertebrates |
| TF331319 | PF01822,CL0164 | ENST00000303746 | Vertebrates |
| TF106506 | PF00023 | ENST00000303941 | Vertebrates |
| TF106401 | PF00249 | ENST00000310806 | Vertebrates |
| TF327329 | PF00051,PF09396 | ENST00000311907 | Vertebrates |
| TF314204 | PF02816 | ENST00000313478 | Vertebrates |
| TF324155 | PF00023 | ENST00000313581 | Vertebrates |
| TF315996 | CL0006 | ENST00000314276 | Vertebrates |
| TF316105 | CL0188 | ENST00000317133 | Vertebrates |
| TF317614 | PF06959 | ENST00000317905 | Vertebrates |
| TF315956 | PF05485 | ENST00000321679 | Vertebrates |
| TF317659 | PF01391 | ENST00000322313 | Vertebrates |
| TF329915 | PF00040 | ENST00000323926 | Vertebrates |
| TF106510 | PF02161 | ENST00000325455 | Vertebrates |
| TF313103 | PF07941 | ENST00000328224 | Vertebrates |
| TF318980 | PF02165 | ENST00000332351 | Vertebrates |
| TF333138 | PF01391 | ENST00000333570 | Vertebrates |
| TF329287 | PF00642 | ENST00000333834 | Vertebrates |
| TF317921 | PF00023 | ENST00000340022 | Vertebrates |
| TF316214 | PF04621 | ENST00000343495 | Vertebrates |
| TF105669 | PF00458 | ENST00000344102 | Vertebrates |
| TF318080 | CL0016 | ENST00000344204 | Vertebrates |
| TF315606 | CL0041 | ENST00000344227 | Vertebrates |
| TF329345 | CL0010 | ENST00000344936 | Vertebrates |
| TF321873 | CL0056 | ENST00000355044 | Vertebrates |
| TF326161 | PF01284 | ENST00000355237 | Vertebrates |
| TF300189 | PF10574 | ENST00000357484 | Vertebrates |
| TF330032 | PF01033 | ENST00000357639 | Vertebrates |
| TF300851 | PF00642 | ENST00000357720 | Vertebrates |
| TF328589 | PF09303 | ENST00000358316 | Vertebrates |
| TF323607 | PF06462 | ENST00000359520 | Vertebrates |
| TF323475 | PF00017 | ENST00000359570 | Vertebrates |
| TF315592 | PF01392 | ENST00000360986 | Vertebrates |
| TF331681 | PF00057 | ENST00000361205 | Vertebrates |
| TF326495 | PF06663 | ENST00000367213 | Vertebrates |
| TF315841 | PF02205 | ENST00000367288 | Vertebrates |
| TF334159 | PF05177 | ENST00000367856 | Vertebrates |
| TF315806 | CL0123 | ENST00000368474 | Vertebrates |
| TF314133 | CL0003 | ENST00000369075 | Vertebrates |
| TF329606 | PF03509 | ENST00000369235 | Vertebrates |
| TF316297 | PF06839 | ENST00000369466 | Vertebrates |
| TF316833 | PF06484 | ENST00000371130 | Vertebrates |
| TF313103 | PF03521 | ENST00000371741 | Vertebrates |
| TF101106 | PF10487 | ENST00000372577 | Vertebrates |
| TF331727 | PF05604 | ENST00000372970 | Vertebrates |
| TF312900 | CL0202 | ENST00000373187 | Vertebrates |
| TF330498 | CL0154 | ENST00000373209 | Vertebrates |
| TF330345 | CL0011 | ENST00000373401 | Vertebrates |
| TF320194 | CL0196 | ENST00000373638 | Vertebrates |
| TF300648 | CL0172 | ENST00000375663 | Vertebrates |
| TF300648 | PF00043 | ENST00000375663 | Vertebrates |
| TF313965 | PF00084 | ENST00000377034 | Vertebrates |
| TF331310 | CL0033 | ENST00000377674 | Vertebrates |
| TF106001 | PF02344,PF01056 | ENST00000377970 | Vertebrates |
| TF313698 | PF03700 | ENST00000380285 | Vertebrates |
| TF315592 | CL0202 | ENST00000380605 | Vertebrates |
| TF324293 | CL0154 | ENST00000380868 | Vertebrates |
| TF316876 | CL0056 | ENST00000380881 | Vertebrates |
| TF332820 | PF08365 | ENST00000381389 | Vertebrates |
| TF329720 | CL0084,PF00533 | ENST00000381989 | Vertebrates |
| TF323983 | CL0179 | ENST00000383733 | Vertebrates |
| TF105391 | CL0128 | ENST00000389202 | Vertebrates |
| TF317067 | CL0266 | ENST00000389247 | Vertebrates |
| TF316056 | PF09004 | ENST00000389568 | Vertebrates |
| TF318198 | CL0188 | ENST00000389821 | Vertebrates |
| TF106341 | PF00010 | ENST00000389936 | Vertebrates |
| TF330156 | PF03815 | ENST00000392504 | Vertebrates |
| TF331707 | CL0219,PF09091 | ENST00000392723 | Vertebrates |
| TF331055 | CL0010 | ENST00000393398 | Vertebrates |
| TF336041 | CL0001 | ENST00000394980 | Vertebrates |
| TF337303 | PF00435 | ENST00000395209 | Vertebrates |
| TF314963 | CL0208 | ENST00000396197 | Vertebrates |
| TF317532 | CL0011 | ENST00000396906 | Vertebrates |
| TF317402 | PF01833,PF01437 | ENST00000397752 | Vertebrates |
| TF106276 | PF08959 | ENST00000398892 | Vertebrates |
| TF331207 | PF00014 | ENST00000399429 | Vertebrates |
| TF106451 | PF07452 | ENST00000204604 | Bilateralia |
| TF314081 | CL0033 | ENST00000215739 | Bilateralia |
| TF313754 | PF00805 | ENST00000221200 | Bilateralia |
| TF331485 | PF00988,CL0014 | ENST00000233072 | Bilateralia |
| TF323999 | PF00773 | ENST00000252889 | Bilateralia |
| TF323502 | PF02185 | ENST00000254260 | Bilateralia |
| TF324918 | PF00057,CL0186 | ENST00000260197 | Bilateralia |
| TF313551 | PF08912 | ENST00000261535 | Bilateralia |
| TF313326 | CL0190 | ENST00000261875 | Bilateralia |
| TF323159 | CL0020 | ENST00000263635 | Bilateralia |
| TF351276 | CL0072 | ENST00000264042 | Bilateralia |
| TF354308 | CL0221 | ENST00000278279 | Bilateralia |
| TF315363 | PF00611 | ENST00000281092 | Bilateralia |
| TF324744 | PF00642 | ENST00000295373 | Bilateralia |
| TF315892 | CL0010 | ENST00000295713 | Bilateralia |
| TF318935 | PF02218 | ENST00000301843 | Bilateralia |
| TF315897 | PF03765 | ENST00000306726 | Bilateralia |
| TF323280 | PF00630 | ENST00000323468 | Bilateralia |
| TF318014 | PF00412 | ENST00000336180 | Bilateralia |
| TF101179 | PF09465 | ENST00000338179 | Bilateralia |
| TF315363 | CL0266 | ENST00000348343 | Bilateralia |
| TF323999 | PF07145 | ENST00000358691 | Bilateralia |
| TF323312 | PF00641 | ENST00000359653 | Bilateralia |
| TF324164 | CL0223 | ENST00000369443 | Bilateralia |
| TF326321 | PF01424,CL0196 | ENST00000371527 | Bilateralia |
| TF324293 | CL0266,PF00621 | ENST00000380868 | Bilateralia |
| TF323674 | PF02825 | ENST00000389044 | Bilateralia |
| TF314351 | CL0126 | ENST00000061240 | AllAnimals |
| TF330032 | CL0263 | ENST00000075322 | AllAnimals |
| TF329240 | CL0200 | ENST00000202017 | AllAnimals |
| TF313988 | PF04707 | ENST00000251170 | AllAnimals |
| TF314316 | PF01463,CL0022 | ENST00000252804 | AllAnimals |
| TF335359 | PF06009 | ENST00000252999 | AllAnimals |
| TF314796 | CL0041 | ENST00000261600 | AllAnimals |
| TF317296 | CL0266 | ENST00000261752 | AllAnimals |
| TF320906 | PF00787 | ENST00000262211 | AllAnimals |
| TF313191 | PF08403 | ENST00000262461 | AllAnimals |
| TF105399 | PF06466 | ENST00000263754 | AllAnimals |
| TF313184 | PF00595 | ENST00000264431 | AllAnimals |
| TF323502 | CL0031 | ENST00000265562 | AllAnimals |
| TF317067 | CL0006 | ENST00000268676 | AllAnimals |
| TF314219 | PF02809 | ENST00000289528 | AllAnimals |
| TF102004 | CL0072 | ENST00000295797 | AllAnimals |
| TF314470 | CL0186 | ENST00000298125 | AllAnimals |
| TF316118 | PF00439 | ENST00000302054 | AllAnimals |
| TF314638 | CL0183 | ENST00000310298 | AllAnimals |
| TF300359 | CL0220 | ENST00000310454 | AllAnimals |
| TF312822 | CL0271 | ENST00000311630 | AllAnimals |
| TF105056 | CL0137 | ENST00000313698 | AllAnimals |
| TF314677 | PF09141 | ENST00000314888 | AllAnimals |
| TF314748 | CL0154 | ENST00000324068 | AllAnimals |
| TF319230 | PF00023 | ENST00000332509 | AllAnimals |
| TF106173 | PF02148 | ENST00000334136 | AllAnimals |
| TF312960 | CL0010 | ENST00000338257 | AllAnimals |
| TF313629 | CL0266 | ENST00000339416 | AllAnimals |
| TF316643 | PF00373 | ENST00000340930 | AllAnimals |
| TF318080 | CL0011 | ENST00000344204 | AllAnimals |
| TF316643 | PF03623 | ENST00000346049 | AllAnimals |
| TF105282 | PF08070 | ENST00000348049 | AllAnimals |
| TF314159 | PF06311 | ENST00000355058 | AllAnimals |
| TF106448 | CL0114 | ENST00000357008 | AllAnimals |
| TF106151 | CL0196 | ENST00000358896 | AllAnimals |
| TF313758 | PF00880 | ENST00000359988 | AllAnimals |
| TF351123 | CL0159 | ENST00000360304 | AllAnimals |
| TF323658 | PF00397 | ENST00000361125 | AllAnimals |
| TF105224 | CL0186 | ENST00000361961 | AllAnimals |
| TF314076 | CL0186 | ENST00000367097 | AllAnimals |
| TF354311 | CL0221 | ENST00000367122 | AllAnimals |
| TF300807 | PF02225 | ENST00000367512 | AllAnimals |
| TF320809 | CL0010 | ENST00000369405 | AllAnimals |
| TF314566 | PF09162 | ENST00000372788 | AllAnimals |
| TF317034 | PF00620 | ENST00000373026 | AllAnimals |
| TF314897 | PF01585 | ENST00000373451 | AllAnimals |
| TF323767 | CL0003 | ENST00000373886 | AllAnimals |
| TF319104 | PF00880 | ENST00000377187 | AllAnimals |
| TF323577 | PF00784 | ENST00000377307 | AllAnimals |
| TF314263 | CL0016 | ENST00000378168 | AllAnimals |
| TF324293 | CL0010 | ENST00000380868 | AllAnimals |
| TF300785 | PF07533 | ENST00000382194 | AllAnimals |
| TF102004 | CL0266 | ENST00000392038 | AllAnimals |
| TF314028 | PF00355 | ENST00000399167 | AllAnimals |
| TF323674 | PF06701 | ENST00000399332 | AllAnimals |
| TF332135 | PF06046 | ENSMUST00000011407 |  |
| TF316484 | PF02140 | ENSMUST00000040422 |  |
| TF316155 | PF02178 | ENSMUST00000040802 |  |
| TF328297 | PF06747 | ENSMUST00000041466 |  |
| TF335390 | PF00096 | ENSMUST00000051869 |  |
| TF329295 | PF00100 | ENSMUST00000084509 |  |
| TF344032 | CL0016 | ENSMUST00000086209 |  |
| TF352132 | PF02415 | ENSMUST00000087258 |  |
| TF335097 | PF00530 | ENSMUST00000090986 |  |
| TF343969 | CL0072 | ENSMUST00000096028 |  |
| TF350794 | PF01352 | ENSMUST00000098508 |  |
| TF327726 | PF08742 | ENSMUST00000098633 |  |
| TF106451 | PF08742,PF01826,PF00094 | ENSMUST00000101614 |  |
| TF313537 | CL0164 | ENSMUST00000102891 |  |
| TF331090 | CL0188 | ENSMUST00000106224 |  |
| TF313147 | CL0202 | ENSMUST00000106949 |  |
| TF334740 | CL0266 | ENSMUST00000109426 |  |
| TF332078 | PF03172 | ENSMUST00000113392 |  |
| TF317514 | CL0069,CL0011 | ENSRNOT00000011676 |  |
| TF101514 | PF08155 | ENSRNOT00000012798 |  |
| TF319471 | PF00096 | ENSRNOT00000034133 |  |
| TF337163 | PF08384 | ENSRNOT00000041557 |  |
| TF335163 | PF03501 | ENSRNOT00000043986 |  |
| TF314473 | CL0010 | ENSXETT00000002556 |  |
| TF336376 | PF10479 | ENSXETT00000010407 |  |
| TF313664 | PF00628,CL0008 | ENSXETT00000017293 |  |
| TF352568 | PF01759 | ENSXETT00000037556 |  |
| TF327588 | CL0291 | ENSXETT00000041950 |  |
| TF343001 | PF06512 | ENSXETT00000045061 |  |
| TF343800 | CL0266 | ENSXETT00000049369 |  |
| TF343807 | PF02135 | ENSXETT00000049701 |  |
| TF330284 | CL0164 | ENSXETT00000055961 |  |
| TF316425 | CL0004 | ENSGALT00000003763 |  |
| TF330943 | CL0102 | ENSGALT00000012528 |  |
| TF343232 | PF00612 | ENSGALT00000017818 |  |
| TF331401 | CL0066 | ENSGALT00000036204 |  |
| TF326300 | PF02181 | ENSDART00000002526 |  |
| TF326300 | PF02205 | ENSDART00000002526 |  |
| TF342779 | PF00681 | ENSDART00000026448 |  |
| TF333311 | PF08344 | ENSDART00000045905 |  |
| TF329914 | CL0184 | ENSDART00000054641 |  |
| TF335519 | PF08441 | ENSDART00000055263 |  |
| TF318964 | CL0266 | ENSDART00000075811 |  |
| TF343508 | CL0016 | ENSDART00000076763 |  |
| TF316498 | PF05485 | ENSDART00000078494 |  |
| TF300180 | CL0202 | ENSDART00000078606 |  |
| TF329039 | CL0188 | ENSDART00000080545 |  |
| TF350019 | PF00260 | ENSDART00000081597 |  |
| TF106435 | PF00748 | ENSDART00000081614 |  |
| TF315837 | PF02188,CL0072 | ENSDART00000084559 |  |
| TF330777 | PF01049 | ENSDART00000086138 |  |
| TF315536 | CL0272 | ENSDART00000087610 |  |
| TF315645 | CL0001 | ENSDART00000097691 |  |
| TF332213 | CL0229 | ENSDART00000098581 |  |
| TF351676 | PF01033 | ENSDART00000104096 |  |
| TF343963 | PF07500 | ENSDART00000036529 |  |
| TF335838 | PF07776 | CG10431-RA |  |
| TF343858 | PF05030 | CG10555-RA |  |
| TF327367 | PF05267 | CG10912-RA |  |
| TF326895 | CL0229 | CG10916-RA |  |
| TF325393 | PF02757 | CG11066-RB |  |
| TF313668 | PF04568 | CG11079-RA |  |
| TF343304 | CL0081 | CG13598-RA |  |
| TF332191 | CL0155 | CG13676-RA |  |
| TF326889 | PF00079 | CG14470-RA |  |
| TF319090 | CL0155 | CG14608-RA |  |
| TF344100 | PF06818 | CG15365-RA |  |
| TF325916 | CL0056 | CG15378-RA |  |
| TF329913 | PF08742,PF00094 | CG15671-RA |  |
| TF350188 | PF02757 | CG15731-RA |  |
| TF324584 | PF00631 | CG15844-RA |  |
| TF351124 | CL0011 | CG16974-RA |  |
| TF316403 | PF01049 | CG17941-RA |  |
| TF321823 | PF00014 | CG18296-RA |  |
| TF312905 | CL0220 | CG31216-RA |  |
| TF323648 | CL0004 | CG32226-RA |  |
| TF343869 | PF03128 | CG32580-RA |  |
| TF343188 | CL0155 | CG32656-RA |  |
| TF351975 | PF00650 | CG32697-RA |  |
| TF319052 | PF05444 | CG34040-RA |  |
| TF319243 | PF00412 | CG4656-RA |  |
| TF343781 | PF01753 | CG4877-RA |  |
| TF315391 | PF07776 | CG5034-RA |  |
| TF313817 | CL0229 | CG5071-RB |  |
| TF316895 | PF00023 | CG5424-RB |  |
| TF343612 | CL0155 | CG5756-RA |  |
| TF327546 | PF05267 | CG5765-RA |  |
| TF313415 | PF10545 | CG6279-RA |  |
| TF105127 | CL0031 | CG7042-RA |  |
| TF313080 | CL0265 | CG7067-RA |  |
| TF336220 | PF00569,CL0220 | CG8529-RC |  |
| TF317532 | PF01753,CL0049 | CG8569-RA |  |
| TF322044 | PF00628 | CG8677-RA |  |
| TF316872 | CL0004 | CG9138-RA |  |
| TF314883 | PF07773 | CG9227-RA |  |
| TF317819 | PF09607 | CG9653-RA |  |
| TF105292 | CL0220 | CG9847-RA |  |
| TF326676 | CL0126 | CG9850-RB |  |
