# Supplementary material for: Quantifying the mechanisms of domain gain in animal proteins
Source: Genome Biol. 2010 Jul 15;11(7):R74. doi: 10.1186/gb-2010-11-7-r74 (PMC2926785; doi:10.1186/gb-2010-11-7-r74)
Supplement: Additional file 6 — Analysis of evidence for retroposition and middle insertions by intronic recombination as mechanisms for domain gain. [file gb-2010-11-7-r74-S6.DOC]

**Possible gains of domains by retroposition and middle gains by intronic recombination**

We further investigated domain gain events that were candidates for gain by retroposition in the human lineage. These were the gain events in which a gained domain was encoded by single exon and a sequence similar to the gained domain was found in another human protein. Retroposition would be supported as a causative mechanism if there were no other exons gained together with the one that encodes the new domain, and also if a long interspersed nuclear element (LINE) retrotransposon was present before the gained domain and/or ‘donor’ domain. Inspection of the candidate domains showed the supporting evidence for the gain of pre-SET and SET domains in the *SETMAR* gene by this mechanism (Figure 4A) but not for other candidate gained domains. However, this inspection was hampered with the fact that the gained domain often existed in multiple copies in the ‘donor’ protein so it was difficult to judge which of the domain repeats was the potential origin. Finally, in the cases where extra exons appeared to be gained with the one that encodes the new domain, retroposition could be excluded as a likely mechanism. The lack of a LINE element does not rule out retroposition as a possible mechanism, rather it does not show additional support for it.

The theory of domain shuffling by intronic recombination states that the exons of the domains inserted into ancestral introns are surrounded by introns of symmetrical phases [40]. We looked at the phases of introns surrounding the domains inserted into the ancestral introns. A list of all intronic gains is in Table S2. 26 of them had the agreeing phases on the boundaries of exons that coded for them, and two more were gained with extra exons that also had agreeing phases on boundaries.

Only one in three possible intron phase combinations gives the same intron phases. We observed a strong bias in agreement of intron phases surrounding the gained domains (57% or 28 out of 49 domains are surrounded with introns of the same phase) and among these an excess of 1-1 phases on exon borders (79% or 22 out of 28). Both symmetrical phases and an excess of 1-1 phases are considered to be supporting evidence for intronic insertions [40]. Moreover, intronic insertions have been shown to be widespread in extracellular matrix proteins and the gained domains in this subset of domains are well known extracellular domains (such as EGF, Sushi, Fibronectin and Immunoglobulin domains) [40].

It has been shown that a class of domains whose borders strongly correlate with their encoding exon borders had experienced significant expansion during animal protein evolution [41]. Moreover, these domains were also found to be frequent in novel metazoan multidomain architectures [42]. It has been hypothesised that these domains have contributed to exon shuffling in metazoa [41,42] and a correlation with symmetrical intron phases surrounding these domains was attributed to their intronic insertions [41]. We were interested to see how well represented these domains were in the set of high confidence domain gain events. We replaced domain identifiers with clan identifiers for these domains, where applicable, and looked at their frequency in the set. We found that they make up about 28% of the set (101 out of 362 gained domains, or 97 out of 333 gain events) which is a significant overrepresentation since only 103 out of total 8,634 domains or clans in the Pfam 23 are in the class of exon-bordering domains (1.2% of all domains). The significant fraction of these domains in our dataset confirms their important role in domain shuffling in metazoa, but the fact that they have been gained about as equally frequently at N- or C-terminus as in the middle of proteins (35, 30 and 32 events, respectively) shows that they have been important not only for intronic gains, but for domain rearrangements in animals in general.
