# Supplementary material for: Quantifying the mechanisms of domain gain in animal proteins
Source: Genome Biol. 2010 Jul 15;11(7):R74. doi: 10.1186/gb-2010-11-7-r74 (PMC2926785; doi:10.1186/gb-2010-11-7-r74)
Supplement: Additional file 7 — Fusion of adjacent genes and NAHR as a mechanism that preceded gene fusions. Discussion of evidence for NAHR as a mechanism that frequently assisted domain gains. [file gb-2010-11-7-r74-S7.DOC]

**Fusion of adjacent genes and NAHR as a mechanism that preceded gene fusions**

Before a domain gain event, domain coding sequence can either exist adjacent to the gene that it will become a part of for a long period of time, or it can exist somewhere else in the genome and domain gain can occur relatively soon after the changes in the genome got the domain into the gene’s proximity. We investigated whether there were instances where a homologue of the gene that had gained a domain, and that was in the same TreeFam family, had a gene coding for the gained domain adjacent to it. We found three cases in the present animal genomes where the gene, homologue of a gene with the gained domain, did not have that domain but was adjacent to the gene which encoded the domain. If these were true separate genes, these would be potential examples for joining of exons from adjacent genes and subsequent gene fusion. However, further inspection showed that they were most likely results of gene annotation discrepancies and were possibly not even true domain gains. These gain events were then excluded from the set of high confidence domain gains (Table S1). These were the following gains: gain of the PF00533 domain in the TF329705 family, of the PF00014 domain in the TF316148 family and of the PF03020 in the TF317729 family.

Next, we investigated whether there was any correlation between domain acquisition and gene duplication. TreeFam trees enable us to distinguish between gene duplication and speciation events. In the entire database, speciation nodes are more frequent than duplication nodes (there are 3.43 times more internal speciation nodes; in total there are 394,853 internal speciation and 115,013 internal duplication nodes). However, in the set of domain gain events that have a human representative for the gain, duplication nodes were more frequent (change in domain architecture was 1.32 times more frequent after gene duplication; 101 gain events occurred after speciation event and 133 after gene duplication). Hence, if we compare the observed frequency of domain gains after duplication relative to after speciation with the expected one we see that after duplication domain gains occur nearly five times more frequently than expected (1.32 relative to 0.29). As a control, we also checked the lengths after speciation and duplication nodes and found that domain gains occurred after every 3,455 units of length when the event was speciation and after 1,274 units of length when the event was duplication. Hence, the lower estimate is that domain gains occur 2.72 (~ 3) times more frequently after gene duplication.

Apart from a duplication of a ‘recipient gene’, domain gains have frequently relied on a duplication of a ‘donor gene’ Moreover, we observed a trend that in the human lineage the younger the gain event was, the more likely it was that the 'donor gene' would be found on the same chromosome (Figure 5). The fact that the tendency was decreasing for the older gains could be related to continuous chromosomal rearrangements. This suggested that the duplication mechanism had favoured creation of duplicates on the same chromosomes. In addition to that, we observed that 'donor genes' were in general found on the same chromosomes as the genes with the gained domains more frequently than would be expected by chance. We calculated this as follows: we compared the number of gains on each chromosome with the number of best hits that we would expect to observe if the duplicates could be inserted equally likely anywhere in the genome (calculated as the portion of the genome length on each chromosome – i.e. individual chromosome length divided by the total length of all autosomes together with X and Y chromosomes - times number of gains on that chromosome). The number of observed 'donor genes' on the same chromosome, 16, is 2.5 times higher than expected 6.5, implying that the mechanism that was creating duplicates had a bias towards the same chromosome.

Taken together, there are several lines of evidence that support NAHR as a plausible important mechanism that mediated the observed domain gains. Firstly, we don’t find that the genes that appear to have been fused in some organisms still exist as adjacent separate forms in others for the majority of the gain events; hence we can assume that often there was a preceding recombination step that initially juxtaposed these genes. Secondly, we see that in the majority of cases at least one of the two genes that took part in the gain event was duplicated beforehand: out of a total of 232 gain events in the human lineage 129 have a possible donor of the gained domain identified and 55 of the remaining ones have gained the domain after an ancestral gene duplication (in total 79% of events). Thirdly, we can discard retroposition as a plausible mechanism that caused these gene/domain duplications. Fourthly, we observe a bias in chromosomal positions of plausible ‘donor genes’ in the way that they are preferentially found on the same chromosomes as genes with gained domains. The bias is more prominent for the younger gain events (Figure 5). Finally, recent work by Kim *et al*. [44] has suggested that even though IR might be important for the formation of new copy number variants in the human genome, NAHR - mediated by Alu elements and existing segmental duplications themselves - had a dominant role in the formation of fixed segmental duplicates.
