# Supplementary material for: Quantifying the mechanisms of domain gain in animal proteins
Source: Genome Biol. 2010 Jul 15;11(7):R74. doi: 10.1186/gb-2010-11-7-r74 (PMC2926785; doi:10.1186/gb-2010-11-7-r74)
Supplement: Additional file 9 — A table that lists domains that are classified as being gained by insertion of new exons(s) into the introns of ancestral genes. [file gb-2010-11-7-r74-S9.DOC]

**Table S2:** **Domains that are gained by insertion of new exons(s) into the introns of ancestral genes.** Phases of introns that surround the exons coding for the gained domains are shown for each gain event. In two cases (marked with * next to domain name) introns surrounding domains did not have symmetrical phases, however additional exons appeared to have been gained together with the one(s) coding for these domains and phases of introns surrounding all inserted exons were symmetrical. It is also noted whether the gained domain(s) is/are coded by single or multiple new exons.

| **TreeFam family** | **Domain gained** | **Phase of**  **5' intron** | **Phase of**  **3' intron** | **Is single exon coding for the gained domain** |
| --- | --- | --- | --- | --- |
|  |  |  |  |  |
| TF336041 | CL0001 | 1 | 1 | Yes |
| TF335097 | PF00530 | 1 | 1 | Yes |
| TF331962 | PF00612* | 1 | 1 | Yes |
| TF313965 | PF00084 | 1 | 1 | Yes |
| TF351678 | CL0202 | 1 | 1 | No |
| TF330156 | PF03815 | 1 | 1 | No |
| TF329915 | PF00040 | 1 | 1 | No |
| TF325130 | PF00023 | 1 | 1 | No |
| TF324293 | CL0010 | 1 | 1 | No |
| TF323674 | PF06701 | 1 | 1 | No |
| TF321873 | CL0056 | 1 | 1 | No |
| TF318080 | CL0011 | 1 | 1 | No |
| TF317532 | CL0011 | 1 | 1 | No |
| TF317402 | CL0159 | 1 | 1 | No |
| TF316484 | PF02140 | 1 | 1 | No |
| TF316380 | CL0011 | 1 | 1 | No |
| TF315592 | PF01392 | 1 | 1 | No |
| TF315592 | CL0202 | 1 | 1 | No |
| TF313537 | CL0164 | 1 | 1 | No |
| TF105391 | CL0128 | 1 | 1 | No |
| TF331319 | PF01822,CL0164 | 1 | 1 | No |
| TF324293 | CL0266,PF00621 | 1 | 1 | No |
| TF314677 | PF09141 | 0 | 0 | No |
| TF314133 | CL0003 | 0 | 0 | No |
| TF314081 | CL0033* | 0 | 0 | No |
| TF313551 | PF08912 | 0 | 0 | No |
| TF300785 | PF07533 | 0 | 0 | No |
| TF106435 | PF00748 | 0 | 0 | No |
| TF325887 | PF10522 | 0 | 1 | Yes |
| TF324610 | PF02732 | 0 | 1 | Yes |
| TF323999 | PF00773 | 1 | 2 | Yes |
| TF322044 | PF00628 | 0 | 1 | Yes |
| TF315892 | CL0010 | 1 | 2 | Yes |
| TF354311 | CL0221 | 2 | 1 | No |
| TF350794 | PF01352 | 0 | 2 | No |
| TF335359 | PF06009 | 1 | 0 | No |
| TF331062 | CL0072 | 2 | 1 | No |
| TF329158 | CL0023 | 2 | 1 | No |
| TF319848 | PF01033 | 1 | 2 | No |
| TF319230 | PF00023 | 2 | 1 | No |
| TF317921 | PF00023 | 2 | 0 | No |
| TF317614 | PF06959 | 0 | 2 | No |
| TF316118 | PF00439 | 0 | 1 | No |
| TF314638 | CL0183 | 2 | 0 | No |
| TF313938 | CL0154 | 0 | 2 | No |
| TF313629 | CL0266 | 0 | 1 | No |
| TF312900 | CL0202 | 1 | 0 | No |
| TF106448 | CL0114 | 0 | 1 | No |
| TF327329 | PF00051,PF09396 | 1 | 2 | No |
