# Supplementary material for: Quantifying the mechanisms of domain gain in animal proteins
Source: Genome Biol. 2010 Jul 15;11(7):R74. doi: 10.1186/gb-2010-11-7-r74 (PMC2926785; doi:10.1186/gb-2010-11-7-r74)
Supplement: Additional file 10 — A table listing significant Gene Ontology terms for human genes that have been extended with a new protein domain during evolution. [file gb-2010-11-7-r74-S10.DOC]

**Table S3: Significant GO terms (P-value < 0.05 after correcting for multiple testing) for human genes that have been extended with a new protein domain.** GO terms are obtained and clustered by using the DAVID service. Abbreviation CC is for Cellular Component, BP for Biological Process and MF for Molecular Function. EASE P-values represent modified Fisher exact P-values. 'Benjamini' shows P-values after applying the Benjamini correction for multiple tests.

|  | **Category** | **GO term** | **EASE P-Value** | **Benjamini** |
| --- | --- | --- | --- | --- |
| Annotation Cluster 1 | CC | basolateral plasma membrane | 1.1 x10^-6^ | 3.1 x10^-4^ |
|  | CC | cell-substrate adherens junction | 4.3 x10^-5^ | 5.8 x10^-3^ |
|  | CC | cell-substrate junction | 6.3 x10^-5^ | 5.8 x10^-3^ |
|  | CC | focal adhesion | 2.3 x10^-4^ | 1.3 x10^-2^ |
|  | CC | adherens junction | 5.9 x10^-4^ | 2.7 x10^-2^ |
|  | CC | anchoring junction | 1.2 x10^-3^ | 4.5 x10^-2^ |
| Annotation Cluster 2 | BP | phosphorus metabolic process | 5.4 x10^-6^ | 9.2x10^-3^ |
|  | BP | phosphate metabolic process | 5.4 x10^-6^ | 9.2x10^-3^ |
|  | MF | adenyl nucleotide binding | 5.6 x10^-6^ | 8.4 x10^-4^ |
|  | BP | post-translational protein modification | 6.2 x10^-6^ | 5.3 x10^-3^ |
|  | MF | purine nucleoside binding | 8.2 x10^-6^ | 7.4 x10^-4^ |
|  | MF | nucleoside binding | 9.7 x10^-6^ | 7.3 x10^-4^ |
|  | MF | ATP binding | 1.5 x10^-5^ | 9.6 x10^-4^ |
|  | MF | adenyl ribonucleotide binding | 2.1 x10^-5^ | 1.2 x10^-3^ |
|  | MF | catalytic activity | 7.5 x10^-5^ | 3.1 x10^-3^ |
|  | BP | protein amino acid phosphorylation | 8.6 x10^-5^ | 3.6 x10^-2^ |
|  | BP | biopolymer modification | 1.1 x10^-4^ | 3.8 x10^-2^ |
|  | BP | protein metabolic process | 1.4 x10^-4^ | 3.4 x10^-2^ |
|  | BP | protein modification process | 2.0 x10^-4^ | 3.7 x10^-2^ |
|  | MF | purine nucleotide binding | 2.7 x10^-4^ | 8.2 x10^-3^ |
|  | MF | protein kinase activity | 5.9 x10^-4^ | 1.4 x10^-2^ |
|  | MF | ribonucleotide binding | 8.0 x10^-4^ | 1.7 x10^-2^ |
|  | MF | purine ribonucleotide binding | 8.0 x10^-4^ | 1.7 x10^-2^ |
|  | MF | protein tyrosine kinase activity | 1.9 x10^-3^ | 3.5 x10^-2^ |
|  | MF | kinase activity | 2.1 x10^-3^ | 3.7 x10^-2^ |
|  | MF | nucleotide binding | 2.2 x10^-3^ | 3.6 x10^-2^ |
|  | MF | transferase activity, transferring phosphorus-containing groups | 2.8 x10^-3^ | 4.0 x10^-2^ |
| Annotation Cluster 3 | MF | zinc ion binding | 7.3 x10^-4^ | 1.6 x10^-2^ |
|  | MF | cation binding | 1.9 x10^-3^ | 3.6 x10^-2^ |
|  | MF | metal ion binding | 2.3 x10^-3^ | 3.6 x10^-2^ |
|  | MF | ion binding | 2.8 x10^-3^ | 4.2 x10^-2^ |
|  | MF | transition metal ion binding | 2.9 x10^-3^ | 4.0 x10^-2^ |
| Annotation Cluster 4 | MF | Ras guanyl-nucleotide exchange factor activity | 2.9 x10^-6^ | 6.5 x10^-4^ |
|  | MF | Rho guanyl-nucleotide exchange factor activity | 6.9 x10^-6^ | 7.7 x10^-4^ |
|  | BP | regulation of Rho protein signal transduction | 5.4 x10^-5^ | 3.0 x10^-2^ |
|  | MF | guanyl-nucleotide exchange factor activity | 2.3 x10^-4^ | 7.2 x10^-3^ |
|  | MF | GTPase regulator activity | 4.1 x10^-4^ | 1.1 x10^-2^ |
|  | MF | nucleoside-triphosphatase regulator activity | 5.1 x10^-4^ | 1.3 x10^-2^ |
|  | MF | small GTPase regulator activity | 1.3 x10^-3^ | 2.6 x10^-2^ |
|  | MF | enzyme regulator activity | 2.3 x10^-3^ | 3.5 x10^-2^ |
| Annotation Cluster 5 | MF | inositol trisphosphate phosphatase activity | 1.9 x10^-4^ | 6.7 x10^-3^ |
|  | MF | inositol-polyphosphate 5-phosphatase activity | 1.9 x10^-4^ | 6.7 x10^-3^ |
| Annotation Cluster 6 | MF | helicase activity | 1.2 x10^-4^ | 4.5 x10^-3^ |
|  | MF | purine NTP-dependent helicase activity | 2.1 x10^-3^ | 3.6 x10^-2^ |
|  | MF | ATP-dependent helicase activity | 2.1 x10^-3^ | 3.6 x10^-2^ |
| Other significant GO terms | MF | scavenger receptor activity | 2.6 x10^-6^ | 1.2 x10^-3^ |
|  | MF | diacylglycerol binding | 3.6 x10^-5^ | 1.8 x10^-3^ |
|  | MF | binding | 6.7 x10^-5^ | 3.0 x10^-3^ |
|  | MF | protein binding | 3.0 x10^-4^ | 8.3 x10^-3^ |
|  | MF | hydrolase activity | 3.1 x10^-3^ | 4.1 x10^-2^ |
|  | BP | cell-matrix adhesion | 1.9 x10^-4^ | 4.0 x10^-2^ |
|  | CC | plasma membrane part | 2.2 x10^-4^ | 1.5 x10^-2^ |
|  | BP | regulation of signal transduction | 1.1 x10^-4^ | 3.2 x10^-2^ |
|  | MF | protein tyrosine kinase activity | 1.9 x10^-3^ | 3.5 x10^-2^ |
